# Supplementary material for: PySTACHIO: Python Single-molecule TrAcking stoiCHiometry Intensity and simulatiOn, a flexible, extensible, beginner-friendly and optimized program for analysis of single-molecule microscopy data
Source: Comput Struct Biotechnol J. 2021 Jul 10;19:4049–58. doi: 10.1016/j.csbj.2021.07.004 (PMC8327484; doi:10.1016/j.csbj.2021.07.004)
Supplement: Supplementary data 1 [file mmc1.pdf]

Supplementary Information

PySTACHIO: Python Single-molecule TrAcking stoiCHiometry Intensity and simulatiOn, a flexible, extensible, beginner-friendly and optimized program for analysis of single-molecule microscopy data

Jack W Shepherd*[1,2], Ed J Higgins*[1,3], Adam J M Wollman[4], Mark C Leake‡[1,2]

[1] Department of Physics, University of York, York, YO10 5DD

[2] Department of Biology, University of York, York, YO10 5DD

[3] IT services, University of York, York, YO10 5DD

[4] Biosciences Institute, Newcastle University, Newcastle, NE1 7RU

‡ To whom correspondence should be addressed. E-mail mark.leake@york.ac.uk

*These authors contributed equally

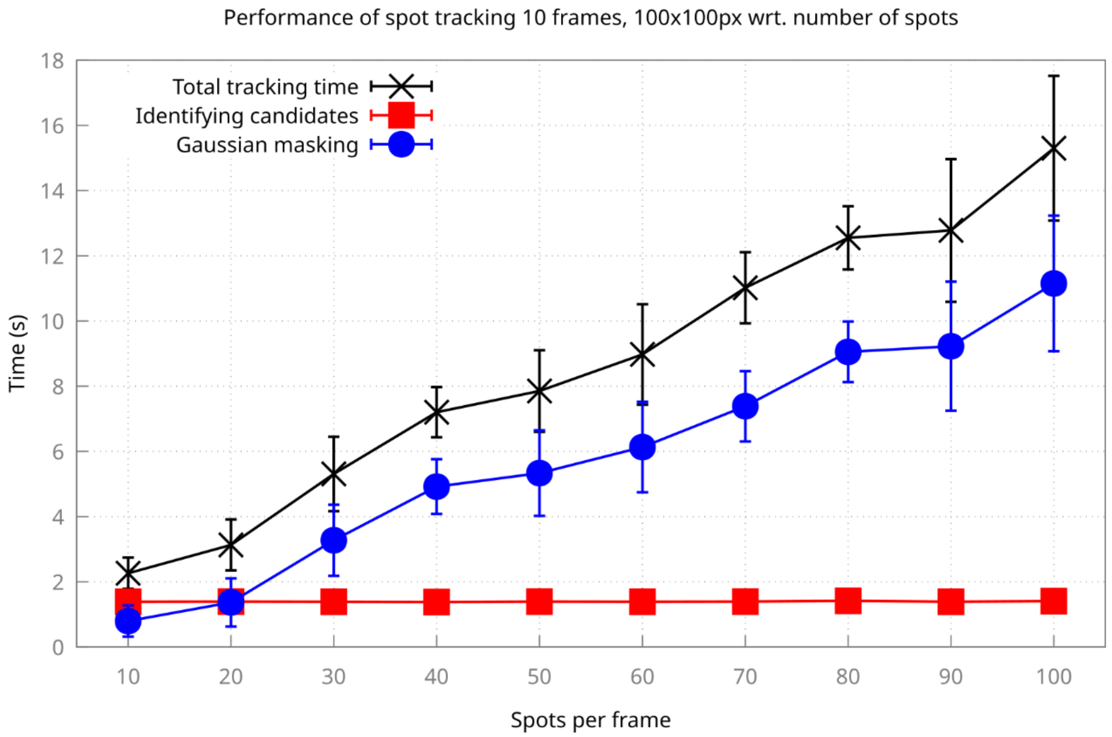

Supplementary Figure 1: Performance of PySTACHIO with increasing spot density (where a "spot" is tracked simulated diffraction-limited fluorescent focus). A simulated frame of 100x100 pixels was simulated with varying numbers of spots. PySTACHIO's performance overall remains almost linear with the majority of the run time being taken up with the iterative Gaussian masking routine.
